# A rare case of mixed mucinous cystadenoma with serous cystadenoma of the pancreas treated by laparoscopic central pancreatectomy

**DOI:** 10.1186/1477-7819-12-318

**Published:** 2014-10-16

**Authors:** Ren-Chao Zhang, Xiao-Wu Xu, Yu-Cheng Zhou, Di Wu, Harsha Ajoodhea, Ke Chen, Yi-Ping Mou

**Affiliations:** Department of General Surgery, Sir Run Run Shaw Hospital, School of Medicine, Zhejiang University, 3 East Qingchun Road, Hangzhou, 310016 Zhejiang Province China

**Keywords:** cystic neoplasm of pancreas, mucinous cystadenoma, serous cystadenoma, laparoscopy, central pancreatectomy

## Abstract

Mixed mucinous cystadenoma with serous cystadenoma of the pancreas is rare. There have been only two previous case reports in the English-language literature. We present a case of a 46-year-old woman who was diagnosed with mixed mucinous cystadenoma with serous cystadenoma of the pancreas. Computed tomography and magnetic resonance imaging showed a cystic neoplasm in the dorsal/proximal body of the pancreas with a clear-margin multilocular cavity and enhanced internal septum. The patient underwent laparoscopic central pancreatectomy. The diagnosis of mixed mucinous cystadenoma with serous cystadenoma of the pancreas was confirmed by pathological examination. The patient was followed up for 3 months and there were no signs of recurrence, or pancreatic exocrine or endocrine insufficiency. To the best of our knowledge, this is the first reported case treated by laparoscopic central pancreatectomy.

## Background

Pancreatic cystic lesions are being detected with increasing frequency due to high-resolution imaging. The prevalence of incidentally detected pancreatic cysts on magnetic resonance imaging is 13.5%, and increases with age [1]. Pancreatic cystic neoplasms have a wide range of diagnostic possibilities, including serous cystic neoplasm, mucinous cystic neoplasm, intraductal papillary mucinous neoplasm and solid-pseudopapillary neoplasm. A mixed mucinous cystadenoma (MCA) with serous cystadenoma (SCA) of the pancreas is rare. There have been only two previous case reports in the English-language literature [2, 3]. We present here a case of mixed MCA with SCA in the dorsal/proximal body of the pancreas. The cystic neoplasm was resected by laparoscopic central pancreatectomy (LCP).

## Case presentation

**Figure 1:**
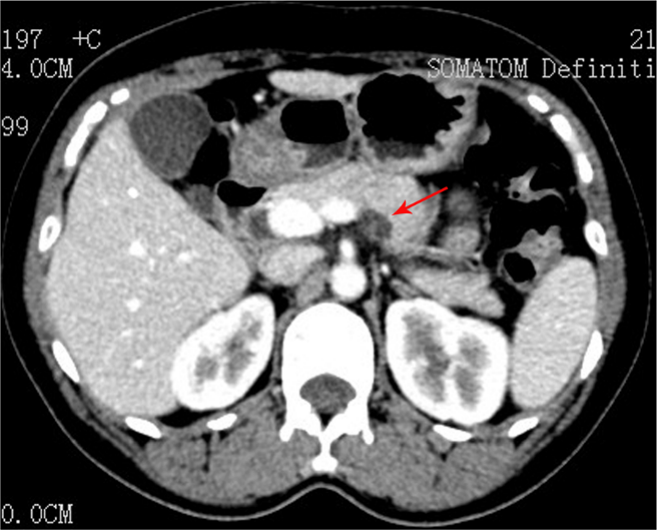
**Computed tomography scan showing a 10 × 14-mm cystic neoplasm (arrow) in the proximal body of the pancreas.**

**Figure 2:**
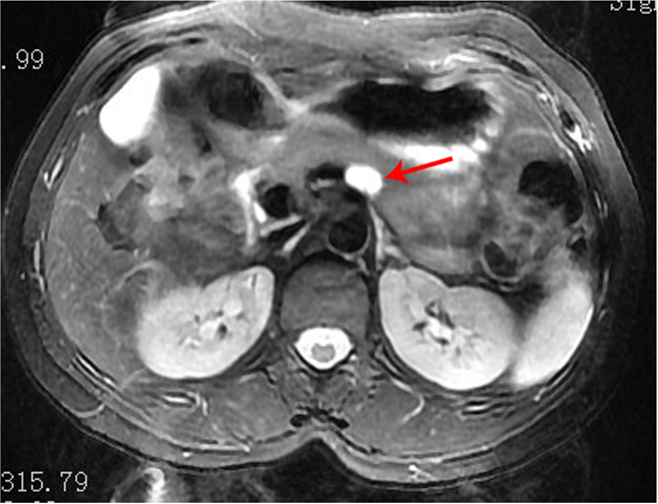
**Magnetic resonance imaging.** There is a 10 × 14-mm cystic neoplasm (arrow) in the proximal body of the pancreas with a clear-margin multilocular cavity and enhanced internal septum.

A 46-year-old woman was admitted to our department because of epigastric dull pain for 1 year. She had no fever, no nausea or vomiting, no diarrhea or melena, and no weight loss. Her past medical and surgical history was unremarkable. The physical examination found no abnormalities. The laboratory tests, including tumor markers (carcinoembryonic antigen (CEA), alpha fetoprotein, carbohydrate antigen 19–9, carbohydrate antigen 724 and carbohydrate antigen 242) were all within normal limits. Computed tomography and magnetic resonance imaging disclosed a 10 × 14-mm cystic neoplasm in the dorsal/proximal body of the pancreas with a clear-margin multilocular cavity and enhanced internal septum (Figures 1 and 2). The cystic neoplasm was deep in the pancreatic parenchyma (less than 3 mm from the main pancreatic duct). From the medical history and the imaging findings, the preoperative diagnosis was a probable cystic neoplasm in the proximal body of the pancreas. Laparoscopic central pancreatectomy was performed.

**Figure 3:**
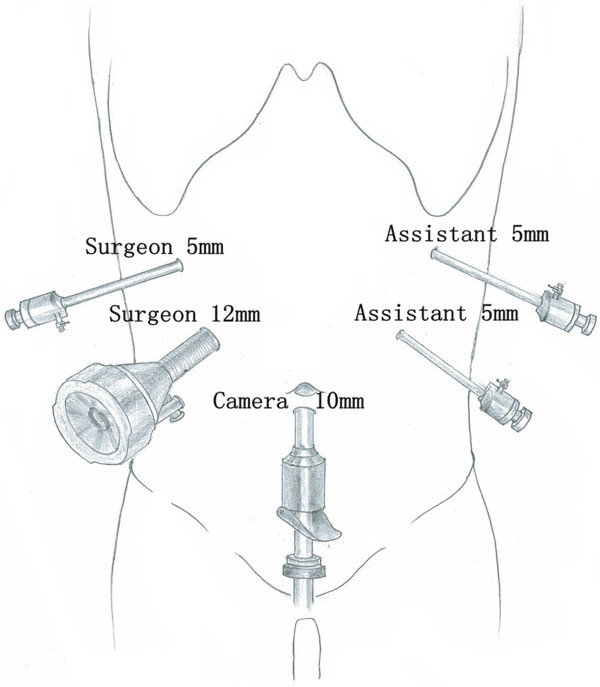
**The location of the trocars.**

The patient was placed in the supine position with her head slightly elevated under general anesthesia. The surgeon and the second assistant, who held the laparoscope, stood on the right side of the patient and the first assistant stood on the left. Carbon dioxide pneumoperitoneum was established (CO_2_ at 15 mmHg) using a Veress needle. One initial 10-mm trocar was placed for the laparoscope below the umbilicus. A 30° telescope was inserted to examine the peritoneal cavity to rule out metastasis disease. After general examination, the other four trocars (one of 12 mm and three of 5 mm) were inserted into the left upper flank, left flank, right upper flank and right flank quadrants; the five trocars were arranged in a V-shape (Figure 3).

**Figure 4:**
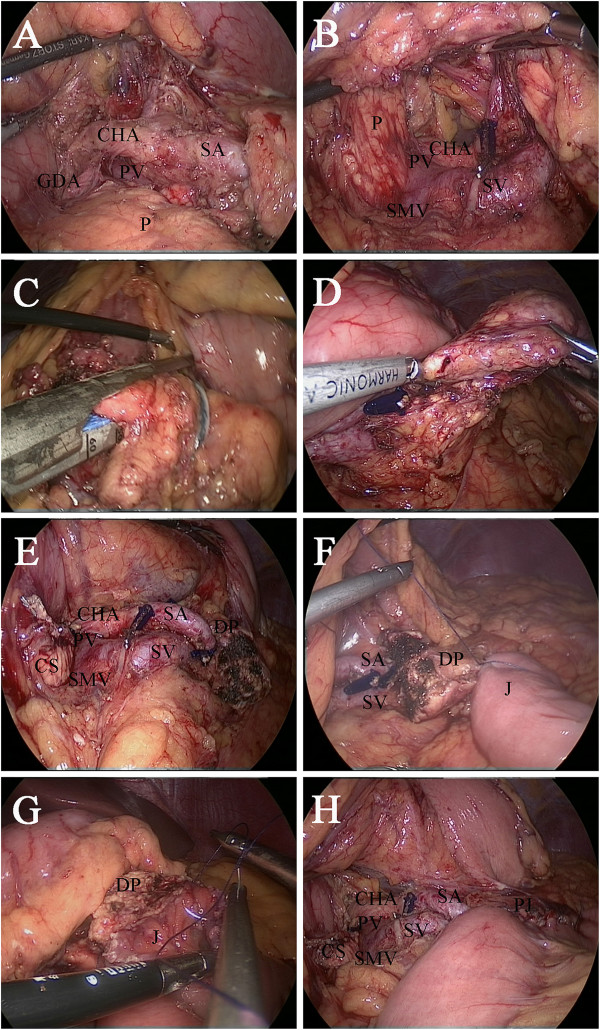
**Laparoscopic central pancreatectomy. (A)** Mobilizing the superior border of the pancreas. **(B)** Mobilizing the inferior border and back of the pancreas. **(C)** Transecting with a 60-mm linear stapler on the right side of the lesion. **(D)** Transecting the distal pancreas. **(E)** View after the central pancreatectomy. **(F)** Suturing the jejunal serosa to the posterior capsule of the pancreas. **(G)** Suturing the full thickness of the jejunum to the pancreas (parenchyma and capsule). **(H)** Final view after reconstruction. CHA, common hepatic artery; CS, cephalic stump; DP, distal pancreas; GDA, gastroduodenal artery; J, jejunum; P, pancreas; PJ, pancreaticojejunostomy; PV, portal vein; SA, splenic artery; SMV, superior mesenteric venous; SV, splenic vein.

The gastrocolonic ligament was divided to give entrance to the lesser sac with a Harmonic scalpel (Harmonic Ace scalpel, Ethicon Endo-Surgery, Inc, Cincinnati, OH). The mobilization of the pancreas began at the superior border until the common hepatic artery and the proximal splenic artery were visible (Figure 4A). Then the pancreas was mobilized at the inferior border to expose the superior mesenteric vein, the splenomesenteric confluence and the portal vein (Figure 4B). After creating a tunnel behind the neck of the pancreas, the pancreas was transected 2 cm proximal to the right side of the tumor with an endoscopic linear stapler (Endocutter 60 staple, blue cartridge; Ethicon Endo-Surgery, Inc, Cincinnati, OH) (Figure 4C). Because of the extent of the pancreatic disease, the central part of the pancreas was dissected from the splenic artery and vein by division of vascular branches with the Harmonic scalpel and vascular clips. The distal transection (2 cm distal to the left side of the tumor) of the pancreas was performed with the Harmonic scalpel (Figure 4D). The frozen section showed a pancreatic benign cystic neoplasm with negative margins.

Next, an end-to-side pancreaticojejunostomy was performed [4, 5]. A row of 3–0 Vicryl suture (coated polyglactin 910 suture, Ethicon Products, Johnson & Johnson, Somerville, NJ) was placed with interrupted stitches between the jejunal serosa and the posterior side of the pancreatic capsule (Figure 4F). The jejunum was opened with the Harmonic scalpel, suitable for distal pancreatic stump. The posterior layer was performed with continuous 3–0 Vicryl suture between the pancreas (parenchyma and capsule) and the full thickness of the jejunum (Figure 4G). The anterior layer was performed in the same way as the posterior layer. Finally, a side-to-side jejunojejunostomy was performed with an endoscopic linear stapler (Endocutter 60 staple, white cartridge). Two drainage tubes were left close to the proximal pancreatic remnant and pancreaticojejunostomy.

The operative time was 240 min and the blood loss was 50 ml. The postoperative course was uneventful. The patient started to take in semi-fluid on day 3 after surgery. The patient was discharged on postoperative day 8. She was followed up 3 months later and there were no signs of recurrence, or pancreatic exocrine or endocrine insufficiency.

**Figure 5:**
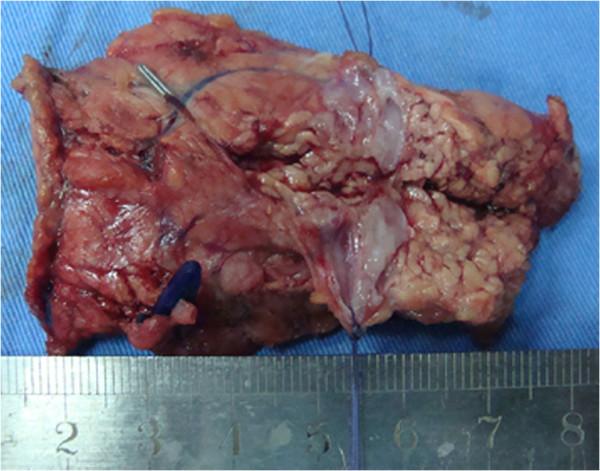
**Resected specimen of the mixed mucinous cystadenoma with serous cystadenoma of the pancreas.**

**Figure 6:**
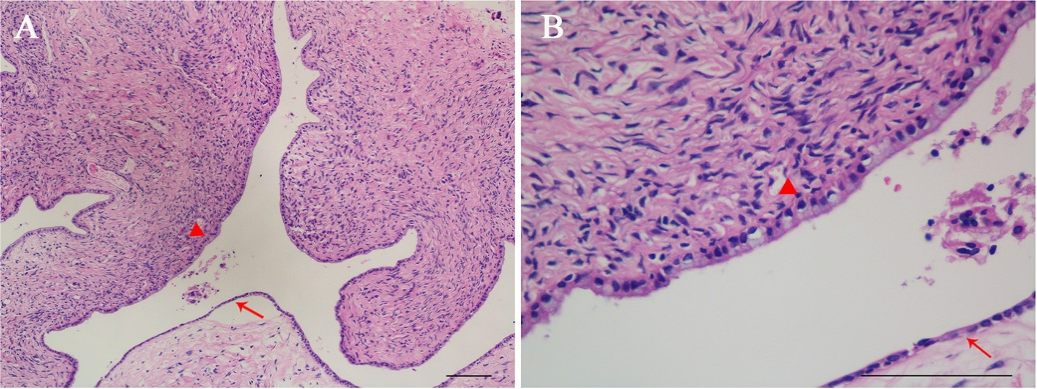
**Histological features of the mixed mucinous cystadenoma with serous cystadenoma of the pancreas.** The arrowhead indicates the mucous columnar epithelium and the arrow indicates the cuboidal epithelium. **(A)** H & E stain, ×100. **(B)** H & E stain, ×400. Scale bars: 100 μm.

The final pathological diagnosis confirmed mixed MCA with SCA of the pancreas. The gross finding was a 15 mm × 15 mm round cystic mass (Figure 5). Furthermore, the cystic mass was multilocular with a fibrous capsule. Microscopically, the cystic wall consisted of fibrous tissues with ovarian-like stroma. The cysts were lined by mucous columnar epithelium or cuboidal epithelium (Figure 6).

## Discussion

The concurrent occurrence of SCA and a variety of primary pancreatic neoplasms, such as pancreatic neuroendocrine tumor, ductal adenocarcinoma, neuroendocrine carcinoma or intraductal papillary mucinous neoplasm, has been reported [6–9]. MCA has also been reported to occur synchronously with a variety of primary pancreatic neoplasms, such as malignant fibrous histiocytoma, anaplastic carcinoma, osteoclast-like giant cell tumor, carcinosarcoma or sarcomatous stroma [10–14]. We reported a rare case of mixed MCA with SCA of the pancreas. To our knowledge, only two cases of mixed SCA with MCA of the pancreas have been reported in the English-language literature [2, 3]. Abe *et al*. [2] reported on a patient who presented with a 6-cm SCA in which the neoplastic cells were interspersed with the neoplastic cells of the MCA in the head of the pancreas. Karidis *et al*. [3] reported on a patient who presented with mixed serous–mucinous cystadenoma at the pancreatic body coexistent with an adenocarcinoma of the pancreatic head.

To date, the origin of mixed MCA with SCA of the pancreas is still unclear. MCA has been thought to arise from duct cells, whereas SCA has been thought to arise from centroacinar cells [15]. Abe *et al*. [2] and Bogomoletz *et al*. [15] reported that the duct cells of the smallest intralobular ducts were adjacent to or imperceptibly merging into the centroacinar cells. The duct cells, centroacinar cells and acinar epithelial cells of the pancreas have a common developmental precursor, which differentiates into each type of stem cell [16]. Therefore, associated oncogenic mutations may occur in the common developmental precursor of the duct cells and centroacinar cells, which leads to mixed MCA with SCA of the pancreas [2].

The American College of Gastroenterology practice guidelines published in 2007 propose that mucinous lesions are characterized by a macrocystic lesion containing viscous fluid rich in CEA, whereas serous lesions have the characteristics of a microcystic morphology with non-viscous fluid and a low level of CEA [17]. Most experts recommend that periodical follow-up for SCA unless the tumor becomes symptomatic and surgical resection for MCA [18]. However, it is difficult to diagnose mixed MCA with SCA of the pancreas before an operation. Including our case, no case has been confirmed before an operation [2, 3]. This interesting phenomenon adds to the challenges in the clinical decision-making in the management of small pancreatic cystic neoplasms. In our case, the patient complained of epigastric dull pain and she was nervous because of the pancreatic cystic neoplasm with an unconfirmed characteristic. After ruling out other diseases and adequate communication with the patient, surgical resection of the pancreatic cystic neoplasm was performed. Finally, mixed MCA with SCA of the pancreas was confirmed by pathological examination. Therefore, evaluation of pancreatic cystic neoplasm should be on a case-to-case basis [18].

Function-preserving pancreatectomy, especially enucleation and central pancreatectomy, is thought to be an ideal procedure for benign or low-grade malignant tumors [19]. Central pancreatectomy is an alternative procedure, if the tumor is close to the main pancreatic duct. It has been reported that LCP for benign or low-grade malignant tumors in the pancreatic neck or proximal body of the pancreas is feasible and safe [20, 21]. However, there are no reports for LCP for mixed MCA with SCA of the pancreas. In our case, the cystic neoplasm of unconfirmed characteristic was located in the dorsal/proximal body of the pancreas and deep in the parenchyma (less than 3 mm from the main pancreatic duct), which contraindicated enucleation or wedge-shaped resection. LCP was an optimal choice of treatment for the patient in whom complicated lymphadenectomy was not necessary. The intraoperative frozen section supported this surgical procedure. Because of the difficulty of identifying an undilated Wirsung duct, an end-to-side pancreaticojejunostomy was executed for the patient without pancreatic fistula. The utility of this method for this case is supported by our successful intraoperative and postoperative results. Compared with open surgery, LCP is associated with lower blood loss and shorter hospital stay, which show its advantages as a minimally invasive operation [21–23]. However, although LCP is a promising procedure, it needs to be validated by more clinical data.

## Conclusions

Mixed MCA with SCA of the pancreas is very rare, and an accurate preoperative diagnosis is difficult. Being aware of the existence of this disease allows for timely management of the pancreatic cystic neoplasm. To the best of our knowledge, this was the first reported case treated by LCP.

## Consent

Written informed consent was obtained from the patient for publication of this case report and any accompanying images. A copy of the written consent is available for review by the Editor-in-Chief of this journal.

## Abbreviations

CEA: carcinoembryonic antigen
H & E: hematoxylin and eosin
LCP: laparoscopic central pancreatectomy
MCA: mucinous cystadenoma
SCA: serous cystadenoma.
